# Multimodal Management of Complex Congenital Heart Disease: A Case of Atrial and Ventricular Septal Defects With a Bifid Apex

**DOI:** 10.7759/cureus.82066

**Published:** 2025-04-11

**Authors:** Ömer Işik, Kardelen Kizilay, Mehmet Dik, Nedret Ülvan

**Affiliations:** 1 Cardiology, Firat University Hospital, Elazig, TUR; 2 Medical School, Faculty of Medicine, Istinye University, Istanbul, TUR; 3 Medical School, Faculty of Medicine, Suleyman Demirel University, Isparta, TUR; 4 Cardiology, Bilkent City Hospital, Cardiology Clinic, Ankara, TUR

**Keywords:** bifid apex, bifid left ventricular apex, congenital heart disease, percutaneous transcatheter closure, structural heart disease

## Abstract

Congenital heart disease (CHD) is one of the most common congenital anomalies influenced by both genetic and environmental factors. Atrial septal defect (ASD) and ventricular septal defect (VSD) are among the most common types of CHD. ASD is more common in females and often remains asymptomatic during childhood, leading to delayed diagnosis. Both surgical and percutaneous closure techniques are used, with evidence supporting early intervention for improved outcomes.

This study presents the case of a female patient with CHD, including ASD, VSD, and a bifid left ventricular apex, who was treated with both surgical and percutaneous approaches. The patient underwent open heart surgery for ASD closure at the age of 17 years. Eight years later, residual ASD and VSD were identified, and percutaneous transcatheter closure was performed using closure devices. Post-procedural imaging confirmed successful defect closure, a significant reduction in left-to-right shunting, and a decrease in right ventricular pressure. However, residual mitral valve prolapse and moderate mitral regurgitation persisted, requiring ongoing monitoring.

The presence of a bifid apex in this patient adds a unique anatomic consideration to managing CHD. While typically considered a benign variant, its association with other structural abnormalities requires careful evaluation. Future management strategies should include detailed imaging studies, such as cardiac MRI, to assess long-term ventricular function and detect potential complications. This case highlights the importance of a comprehensive, multimodal management strategy that includes surgical and transcatheter techniques for complex coronary artery disease cases. Regular follow-up with echocardiography and cardiac MRI is essential to monitor residual defects, assess ventricular function, and ensure long-term treatment success. Early detection and timely intervention are critical to optimizing patient outcomes, preventing complications, and improving quality of life.

## Introduction

Congenital heart disease (CHD) is a congenital anomaly that can occur at any age, and its genetic factors are still being studied [1]. Although the exact causes of CHD are not fully understood, it remains the most common congenital anomaly in newborns and young adolescents [2]. Advances in neonatal care, particularly in the management of premature infants and early intervention, have significantly improved the survival of individuals with CHD, which, in turn, has affected its prevalence. CHD is now estimated to occur in approximately 1% (~0.8%) of live births, with an increasing number of patients living into adulthood due to improved medical care. Environmental factors such as maternal infections, teratogens, and conditions such as maternal diabetes have been identified as contributing to the development of CHD [3]. A bifid apex is a rare congenital heart anomaly that is typically associated with other congenital anomalies, although it can occasionally occur in isolation. There is limited information in the literature regarding this anomaly.

This malformation is rarely observed in otherwise normal human hearts or in association with congenital heart defects. Interestingly, a bifid apex is found in the normal hearts of marine mammals such as whales, dugongs, and manatees. This adaptation is thought to help these marine mammals cope with the changes in pulmonary circulation associated with diving.

Atrial septal defect (ASD) is the third most common type of CHD, and secundum ASD is the most common subtype. This defect causes a left-to-right shunt that can lead to progressive right atrial and ventricular enlargement over time. ASD is more common in females and tends to be diagnosed later in life, often after infancy [4]. The presence of an ASD can lead to long-term complications such as heart failure or arrhythmias if left untreated.

Ventricular septal defect (VSD), another common form of CHD, is classified based on its location in the septum. VSDs can be classified as perimembranous or muscular, and their size and location determine the clinical management. Smaller VSDs may close spontaneously or remain hemodynamically insignificant, whereas larger defects may require surgical intervention.

In this study, we evaluated a female patient with CHD who underwent both ASD and VSD closure. The first surgery, an open heart procedure, was performed in 2005 and focused on ASD closure only. The second procedure, performed eight years later, involved closure of both the ASD and VSD using a percutaneous device. This case highlights the importance of long-term surveillance in patients with CHD, as well as the complexity of managing additional anomalies such as a bifid apex. The use of multimodal interventions, including open heart surgery and percutaneous closure techniques, underscores the evolving treatment strategies for complex congenital heart defects.

## Case presentation

A patient born at 38 weeks and 3 days with no prenatal fetal cardiac follow-up was diagnosed with a pathologic murmur on physical examination after birth. Echocardiography revealed a VSD, ASD, and a double-chambered right ventricle. As the patient’s initial echocardiographic measurements were not available, the exact sizes of the ASD and VSD were unknown.

**Figure 1:**
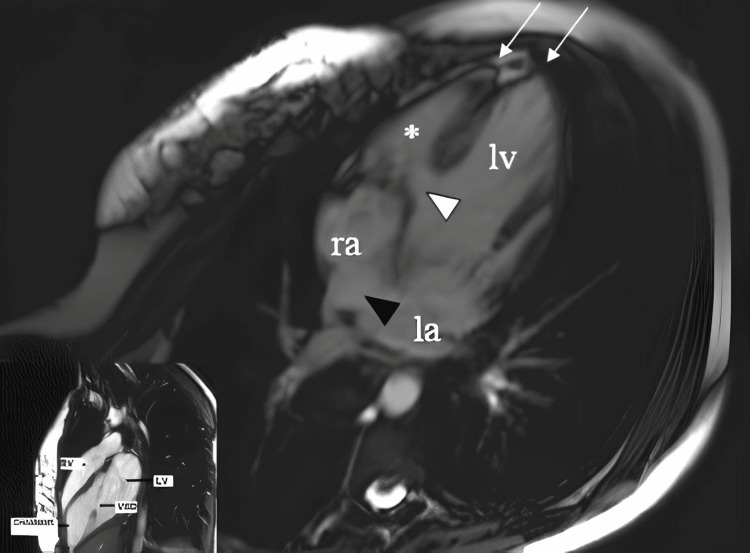
Cardiac MRI showing atrial septal defect (black arrow) and ventricular septal defect (white arrow). The image also reveals a bifid left ventricular apex, suggesting a rare structural abnormality contributing to the patient’s condition.

The patient was followed routinely by the pediatric cardiology service. The patient had no cardiac complaints until the age of 17 years. When the patient was admitted to the cardiology clinic for exertional dyspnea that began at the age of 17, cardiac auscultation revealed a systolic murmur at the left upper sternal border. Echocardiography showed an 18 mm wide VSD, a small ASD, biventricular hypertrophy, mitral valve prolapse (MVP), and a double-chambered right ventricle. The patient’s cardiac MRI showed a VSD, ASD, and bifid ventricular apex (Figure 1) [4].

**Figure 2:**
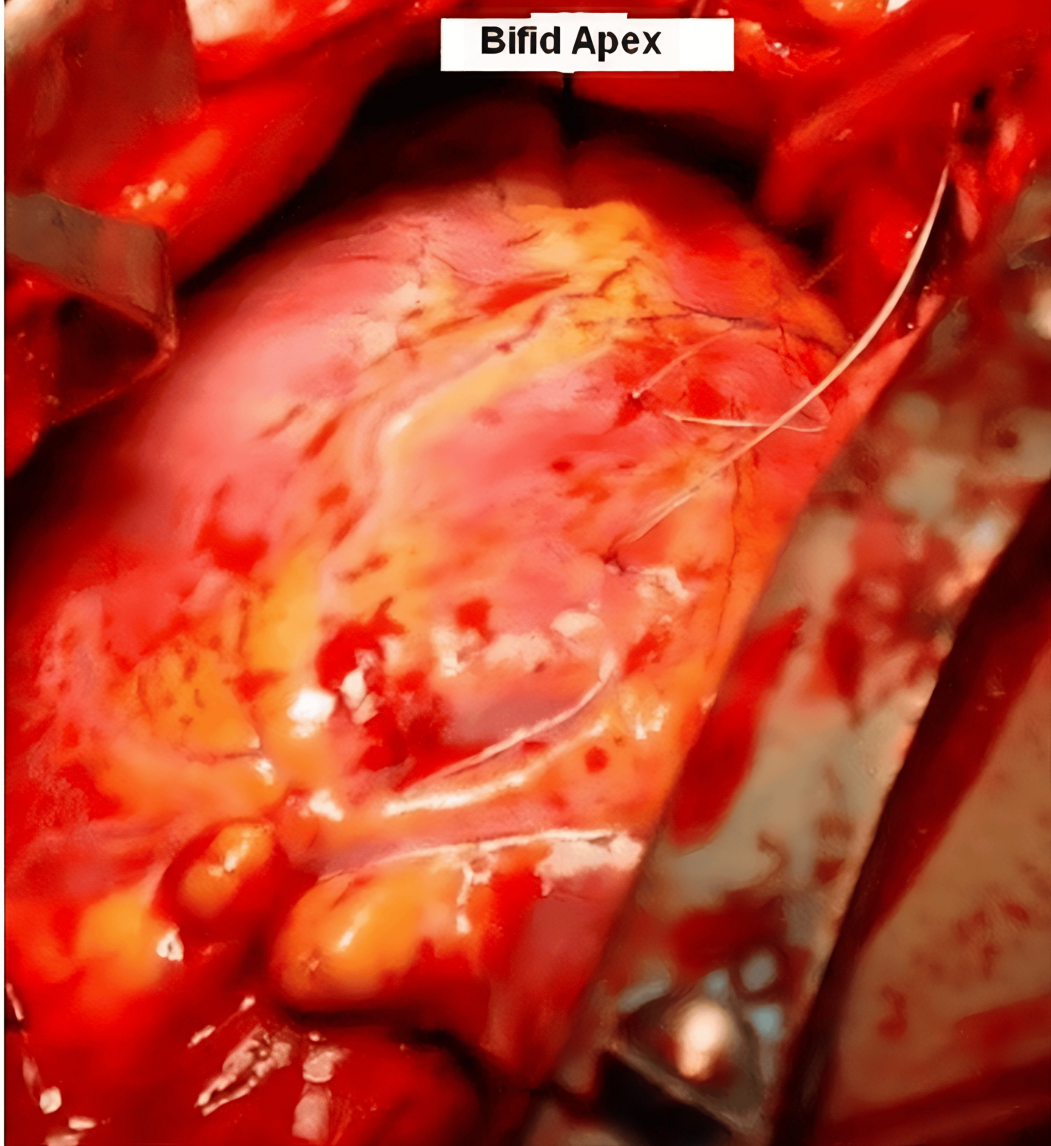
Intraoperative findings showing a bifid left ventricular apex with a 2 cm deep cleft, an uncommon congenital structural variation. This anomaly was identified during surgical repair of the atrial and ventricular septal defects.

Before the planned surgical intervention, a diagnostic right heart catheterization was performed, during which the interventricular pressure gradient was recorded at 90 mmHg (normal: <30 mmHg), prompting the decision to proceed with surgery. The patient was scheduled for surgery. During surgery, it was noted that the cardiac anatomy was a bifid apex and there was a 2 cm deep cleft. The intraoperative findings were inconsistent with the preoperative echocardiographic and MRI results. The heart size was smaller than expected, and the pulmonary arteries appeared normal-sized. The right ventricular volume was reduced, and the tricuspid valve had a smaller diameter. No VSD was identified. Normal saline was flushed through the left ventricle, but no outflow through the right ventricle was observed. On examination, the pulmonary arteries were normal and the right ventricular outflow tract was free of obstructive lesions. In addition, the ASD was partially closed due to the reduced right ventricular volume (Figure 2) [4,5].

The size of the ASD was measured to be 15 mm, and the ASD was closed during surgery. Her oxygen saturation was 92%-93% in the operating room before anesthesia and 96%-97% on postoperative day seven.

The patient, who continued routine follow-up and did not receive any medical treatment, had a secundum ASD (16 mm), bifid left ventricle (interventricular septum to the posterior of the tricuspid valve, a muscular aneurysmatic structure, and a VSD with a 120 mmHg gradient on continuous-wave Doppler through a thin tunnel), MVP, and second-degree mitral regurgitation (MR) on echocardiography performed approximately six years after surgery. Cardiac MRI findings were similar. Diagnostic cardiac catheterization for coronary artery disease was performed, and the diagnosis was confirmed.

As the patient had a transischemic attack (TIA) and no arrhythmia was detected in the ECG/rhythm Holter follow-up, it was decided to percutaneously close the ASD and VSD. Percutaneous transcatheter closure of the VSD and ASD with closure devices was performed the day after admission. After percutaneous closure, the patient was placed on antiplatelet therapy for six months. On transthoracic echocardiography, the right ventricle was hypoplastic in four chambers, and, in short-axis sections, inlet-outlet and apex formation were observed. Left ventricular septal movements were slightly paradoxical, and the ejection fraction (EF) was 55%. The EF did not decrease. The septal occluder device position on the atrial septum was good, and there was no remnant. Similarly, there was no muscular VSD occluder remnant. The mitral valves were seen to prolapse toward the left atrium in systole. The left ventricle was seen to continue anteriorly to the right with a broad muscular tissue (bifid left ventricle) posterior to the left ventricle tricuspid valve. Percutaneously closed muscular VSD, secundum ASD, and mid-MR were also observed. Estimated right ventricle systolic pressure measured at the tricuspid valve was 18 mmHg. The left atrial anteroposterior diameter was measured as 4.4 cm,, and the left atrium-to-aortic root ratio was measured as 1.3. This ratio indicates that the second operation was effective. The last echocardiogram showed a bifid left ventricle, percutaneously closed muscular VSD, secundum ASD, MVP, and moderate-to-severe MR.

The patient, who did not receive medical treatment, was followed up with atrial tachycardia attacks on ECGs obtained several times because of palpitations. Only one of the attacks required intervention, and the others resolved spontaneously. An electrophysiology study/ablation procedure was planned in case of recurrence of the attacks and symptoms. The patient, whose resting heart rate is 50-60 beats/minute, is continuing follow-up without medical treatment. Our patient is currently asymptomatic, with no deterioration in left ventricular ejection fraction, and left ventricular end-systolic diameter remains non-dilated. The patient is followed up with cardiologic examinations and transthoracic echocardiogram every six months. If the end-systolic diameter increases, the ejection fraction decreases, or arrhythmic events occur during follow-up, the heart team will discuss the surgical indication for valve replacement.

## Discussion

CHD includes some disorders before birth, which are based on the interaction of some genetic or environmental factors during the formation of the heart [6]. One of the most common CHDs is ASD, which is more common in women [7]. This case presents a complex scenario of CHD, highlighting the challenges in managing combined ASD and VSD with additional anomalies such as a bifid left ventricular apex and MVP. Despite significant advancements in diagnostic and interventional cardiology, these cases emphasize the importance of individualized treatment planning and long-term follow-up.

The initial presentation of small VSD and ASD, combined with the findings of a double-chambered right ventricle, underscores the variability in the progression of CHD. The lack of symptoms until the age of 17, when exertional dyspnea developed, reflects the insidious nature of CHD progression. Over time, the defects led to significant structural and functional alterations, including biventricular hypertrophy and elevated pulmonary artery pressure, necessitating surgical intervention.

The first surgical intervention aimed to address the larger defect, the ASD, leaving the VSD untreated, as it was deemed clinically insignificant at the time. This decision underscores a common challenge in CHD management, i.e., determining the appropriate timing and extent of intervention to balance surgical risks with long-term outcomes. However, the progression of the defects over six years, including the development of a significant VSD and a recurrent ASD, necessitated a second intervention.

The bifid left ventricular apex and the presence of a cleft add unique anatomical considerations. These findings are rare and require meticulous evaluation to differentiate between structural anomalies and pathological processes. The successful use of percutaneous closure devices for both the ASD and VSD in the second surgery demonstrates the evolution and efficacy of minimally invasive techniques in treating complex CHD cases.

Following the second intervention, the patient’s left ventricular ejection fraction was reduced to 30%, and paradoxical septal motion was observed. These findings raise concerns about potential complications, such as altered myocardial mechanics due to the placement of occlusion devices or pre-existing myocardial dysfunction. Additionally, the persistence of MVP and moderate MR reflects the ongoing burden of valvular pathology, which may contribute to symptoms such as palpitations and require further management.

The residual left-to-right shunt observed on follow-up imaging, despite effective occlusion of the primary defects, highlights a common limitation of interventional procedures. Signal loss consistent with residual shunting indicates the potential for device-related leaks, which may have implications for right ventricle and pulmonary artery pressures over time. However, the significant reduction in shunting and normalization of pulmonary artery pressures postoperatively are promising outcomes.

This case underscores the necessity of long-term follow-up in CHD patients, particularly those with complex anatomical and functional anomalies. Regular imaging, including advanced modalities such as cardiac MRI, plays a crucial role in monitoring device integrity, residual defects, and ventricular function.

The management of residual defects, persistent valvular abnormalities, and myocardial dysfunction remains an area for further investigation. Advanced imaging techniques and computational modeling may provide insights into optimizing device placement and predicting long-term outcomes. Furthermore, emerging technologies such as artificial intelligence could play a role in improving diagnostic precision and individualizing treatment strategies.

## Conclusions

This case underscores the importance of long-term surveillance and timely intervention in patients with CHD. Despite successful closure of ASD and VSD via percutaneous techniques, residual MR and atrial tachycardia persist, highlighting the complexity of managing congenital defects, particularly when additional anatomical anomalies such as a bifid left ventricle are present. These findings emphasize the need for close, ongoing follow-up with echocardiography and cardiac MRI to monitor for potential complications such as arrhythmias, ventricular dysfunction, and the progression of MR. The patient’s clinical stability post-procedure, without significant deterioration in left ventricular ejection fraction or end-systolic diameter, suggests that the condition is currently under control. However, careful monitoring is crucial, as any future changes in ventricular function or arrhythmic events may require further interventions, including potential surgical considerations for valve repair or replacement. The use of percutaneous closure techniques in this case has contributed to a reduction in surgical burden, improved recovery times, and provided an effective means of managing complex congenital defects with fewer long-term risks associated with open heart surgery. These advancements in minimally invasive procedures continue to offer significant benefits in treating CHD, enhancing both immediate outcomes and the patient’s quality of life. Continued research and innovation in percutaneous techniques will further improve outcomes for patients with complex congenital heart conditions.
